# 3D-GNOME 3.0: a three-dimensional genome modelling engine for analysing changes of promoter-enhancer contacts in the human genome

**DOI:** 10.1093/nar/gkad354

**Published:** 2023-05-09

**Authors:** Michal Wlasnowolski, Michal Kadlof, Kaustav Sengupta, Dariusz Plewczynski

**Affiliations:** Faculty of Mathematics and Information Science, Warsaw University of Technology, Warsaw, 00-662, Poland; Centre of New Technologies, University of Warsaw, Warsaw, 02-097, Poland; Faculty of Mathematics and Information Science, Warsaw University of Technology, Warsaw, 00-662, Poland; Faculty of Mathematics and Information Science, Warsaw University of Technology, Warsaw, 00-662, Poland; Centre of New Technologies, University of Warsaw, Warsaw, 02-097, Poland; Faculty of Mathematics and Information Science, Warsaw University of Technology, Warsaw, 00-662, Poland; Centre of New Technologies, University of Warsaw, Warsaw, 02-097, Poland

## Abstract

In the current update, we added a feature for analysing changes in spatial distances between promoters and enhancers in chromatin 3D model ensembles. We updated our datasets by the novel *in situ* CTCF and RNAPII ChIA-PET chromatin loops obtained from the GM12878 cell line mapped to the GRCh38 genome assembly and extended the 1000 Genomes SVs dataset. To handle the new datasets, we applied GPU acceleration for the modelling engine, which gives a speed-up of 30× versus the previous versions. To improve visualisation and data analysis, we embedded the IGV tool for viewing ChIA-PET arcs with additional genes and SVs annotations. For 3D model visualisation, we added a new viewer: NGL, where we provided colouring by gene and enhancer location. The models are downloadable in mmcif and xyz format. The web server is hosted and performs calculations on DGX A100 GPU servers that provide optimal performance with multitasking. 3D-GNOME 3.0 web server provides unique insights into the topological mechanism of human variations at the population scale with high speed-up and is freely available at https://3dgnome.mini.pw.edu.pl/.

## Graphical Abstract

**Graphical Abstract:**
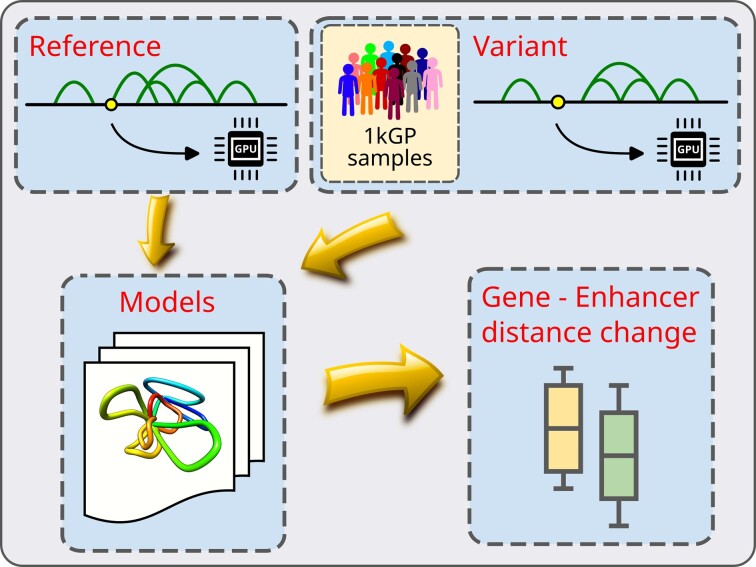
Below we attached the caption for the graphical abstract:3D-GNOME 3.0: a three-dimensional genome modelling engine for analysing changes of promoter-enhancer contacts in the human genome.

## INTRODUCTION

**Figure 1.**
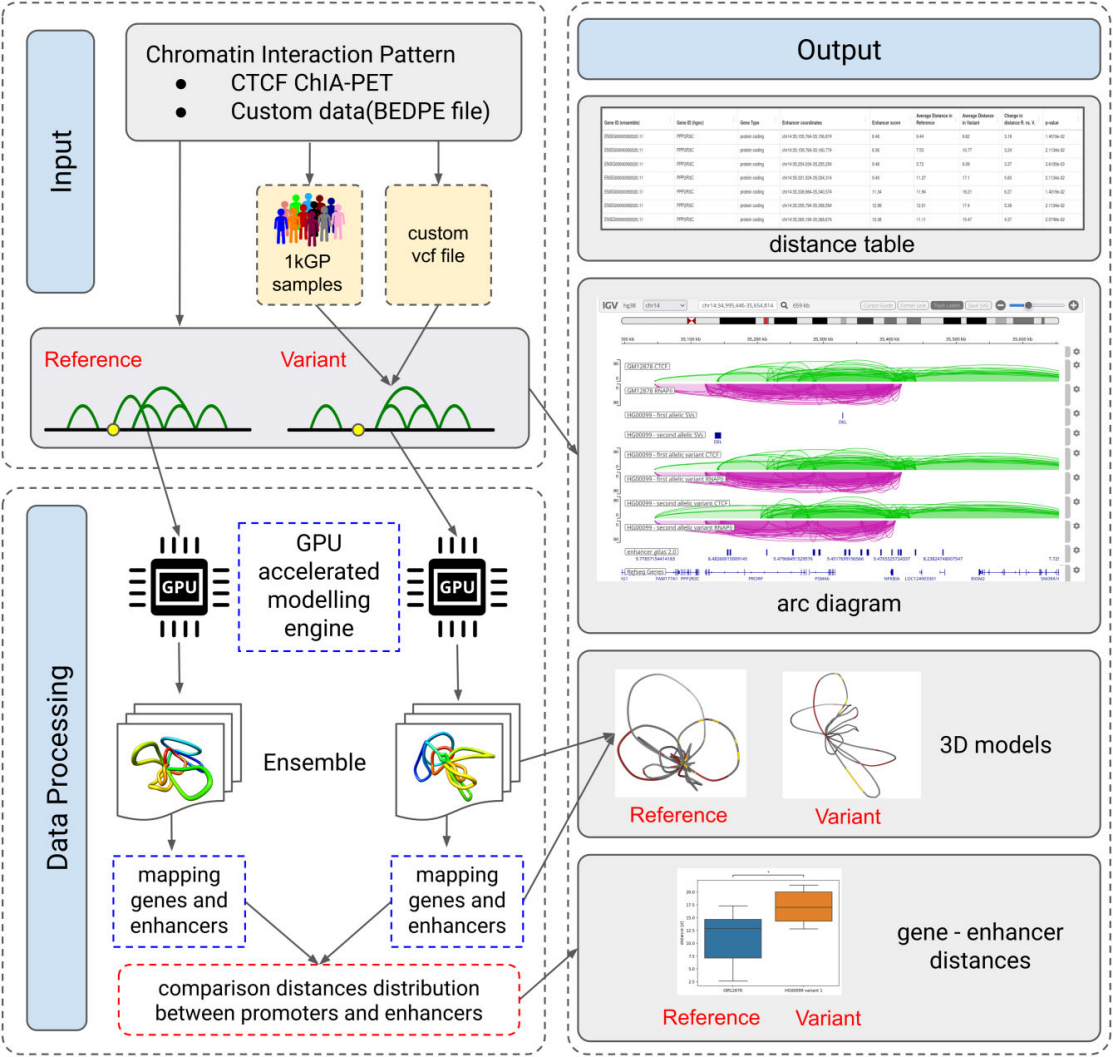
Scheme of 3D-GNOME 3.0 server architecture.

One of the primary challenges in human genetics, precision medicine, and evolutionary biology is deciphering gene expression regulation and understanding the transcriptional effects of genome variation (1–3). The three-dimensional organisation of chromatin and the spatial proximity between enhancers and gene promoters have been shown to impact gene expression significantly (4–7). Additionally, structural variants (SVs) that alter chromatin structure can profoundly affect gene regulation (8). Genomic studies indicate that SVs can directly impact the interactions between the promoter and enhancer regions of the chromatin (9,10), which could lead to the development of new therapeutic targets and diagnostic tools (11,12). Thus, developing an accessible tool to investigate these complex interactions is essential for advancing the understanding of gene expression regulation and genome variation. This paper proposes a new version of the 3D-GNOME web server (13,14) that provides tools for comparing different 3D structures of the genome (Figure 1). It enables the analysis of changes in the modelled distance distribution between enhancers and gene promoters in the GM12878 cell line, with genomic rearrangements based on Structural Variants (SV) provided by 1000 Genome Project (15). Furthermore, although the primary focus of the 3D-GNOME web server is to analyse large-scale genome rearrangements, such as Structural Variants, it is also capable of handling small changes like insertions or deletions of a few nucleotides (indels), particularly if they have an impact on chromatin contacts. By offering differential contact sets from various samples, the system allows for a broader range of structural polymorphisms to be analysed. In both cases, the server allows for the provision of custom variants and chromatin interaction datasets.

The previous version of the 3D-Gnome web server (2.0) (13) was developed by incorporating Structural Variants to model 3D structural changes in chromatin, based on long-read ChIA-PET GRCh37 data and SVs from 1000 Genome Projects. With the update to both: 3D data into *in situ* ChIA-PET GRCh38 and the new SV dataset from 1kGP, CPU-based computation turned out to be slow. Therefore, to handle new dense datasets, we extended the 3D-GNOME modelling engine (which is described in detail in (16)) with GPU-acceleration. Moreover, because of up to 30× speed-up achieved now, we can create ensembles of 3D models of reference and reordered genomes to study the changes in the promoter-enhancer modelled distance distribution, providing a more specific picture to understand gene expression.

Apart from the major changes described above, we shifted the web server to new GPU servers with A100 graphics cards, giving users fast results. Moreover, to make the results more comprehensible, we changed the loop viewer to IGV (17) and the model viewer to NGL (18). Also, for better visualisation of the modelling results, models are coloured based on gene promoter body and enhancer location.

As far as we are aware, only a limited number of web servers are available that offer the ability to generate chromatin 3D models and detailed genomic feature analysis (19,20). However, none of these web servers provides the option to calculate changes in spatial distances between enhancers and genes caused by structural variants in different human populations by generating full ensembles of chromatin 3D models based on high-resolution ChIA-PET data, which is why we find our new feature unique. This new release gives abilities for analysing the potential impact of genome spatial changes on gene activity, allowing for a deeper understanding of gene regulation and cellular processes.

## NEW FEATURES AND UPDATES

### New datasets

In the previous version of 3D-GNOME web server, the modelling of chromatin structure was based on long-range ChIA-PET data, including CTCF and RNAPII chromatin interactions of the GM12878 cell line mapped onto the GRCh37 reference genome, as well as structural variants from 2502 samples from the 1000 Genomes Project release 3, also mapped onto GRCh37.

In the current version, we have replaced the previous dataset of CTCF and RNAPII interactions in the GM12878 cell line, which was obtained from *long-read* ChIA-PET (21,22), with high-resolution data from *in situ* ChIA-PET (23), which was mapped onto the GRCh38 reference genome. The new dataset provides substantially more chromatin interactions with higher confidence and offers a more comprehensive and accurate view of the genome's spatial architecture in the GM12878 cell line. As a result of this new dataset, the quality of chromatin 3D models generated using 3D-GNOME has also improved.

The structural variant dataset (15) has been updated, with the previous GRCh37 version replaced with a GRCh38 version. The number of samples expanded to 3202 by including 30x high-coverage data from the NYGC on GRCh38. These updates provide a more comprehensive and accurate representation of chromatin structure, enabling further analysis and understanding of its impact on gene expression.

### GPU-accelerated modelling engine

We have implemented GPU acceleration into our modelling engine, which is based on the Simulated Annealing Monte Carlo method, to address the significant increase in calculation time when analysing ensembles of chromatin 3D models using much larger datasets of chromatin 3D contacts. As a result, we have achieved a 30x speed-up compared to the previous version. To facilitate subsequent analysis, we have converted the models from the hcm, 3D-GNOME native format to the XYZ and mmCIF formats, which can handle models with many more beads than the PDB format.

### Updated web server architecture

The primary modelling task is performed on the Eden cluster, an in-house heterogeneous computing cluster equipped with Nvidia DGX A100 nodes. The Eden cluster is controlled by the Slurm (24) queuing system, which is deployed at the Faculty of Mathematics and Information Science at Warsaw University of Technology. The 3D-GNOME web interface runs on an LXC container in a ProxMox environment.

When a user submits a modelling request, the Flask web server executes a sequence of tasks, including validating the data, saving the data in a shared location with the Eden cluster, creating a database entry for the new task, and passing the task identifier to a concurrently running Gnu Parallel process (25). Gnu Parallel runs a Python script with a pipeline that performs local data pre-processing and then sends a request to run the modelling on the cluster.

Communication between the container and the Slurm controller is done through a REST API. The pipeline process periodically checks the status of the Slurm task, and when it receives information about the completion of the computation, it performs post-processing and updates the database entry. Once the modelling is complete, the user can view the results by refreshing the page.

### Ensemble analysis

A key feature of the current update is the ability to analyse changes in spatial distances between gene promoters and enhancers caused by structural variants. This involves generating multiple chromatin 3D models for a specific chromatin region, both for the reference chromatin contact pattern and for the pattern affected by the SVs. Genes (GRCh38) and enhancers (based on Enhancer Atlas 2.0 (26), liftovered to GRCh38) are mapped onto each model, and the Euclidean distance between enhancers is calculated. The distance measure is specific to the 3D-GNOME engine, so the key factor for analysis is a change in distance distribution, as demonstrated in Sadowski *et al.* (27). To test the significance of the change in distance distribution, we use the Mann–Whitney *U* test with a *P*-value threshold of 0.05. This analysis provides insights into the impact of SVs on gene regulation by identifying changes in the spatial proximity between gene promoters and enhancers.

### Input

In the request form, as in the previous version, the user may use prepared datasets for GM12878 chromatin interactions, set the region of interests and 3D modelling parameters and choose the sample ID of structure variation from the 1000 Genome Project database (15). It is also possible to upload chromatin interactions in BEDPE format or SVs in VCF format (VCFv4.2).

In the current version, we add to the form checkbox that runs ensemble analysis and sets the number of models in the ensemble.

### Output

The 3D-GNOME web server presents new results in a fully responsive table and a boxplot generator for visualising the distribution of gene-enhancer distances. In addition, the web server has been updated with new tools for data visualisation, building on the functionality of the previous version.

**Figure 2.**
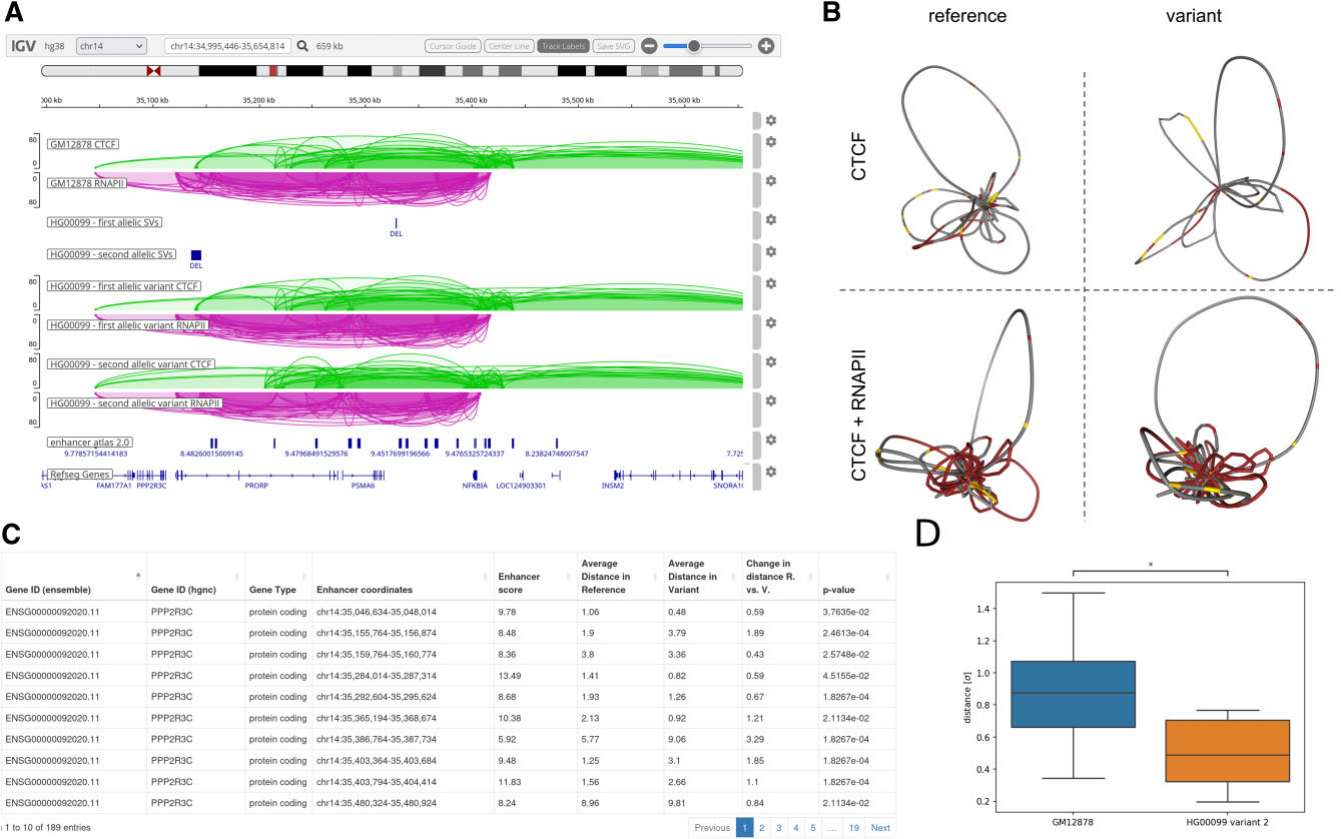
Results for the region chr14:35605439–35615196: (**A**) Screenshot of the IGV browser showing CTCF-mediated (green) and RNAPII-mediated (purple) chromatin interactions, as well as genomic annotations for the selected region. Tracks 1–2 display arcs for the reference cell line (GM12878), tracks 3–4 show SVs mapped to the reference genome, and tracks 5–8 show the arcs for variant I and variant II, respectively. Track 9 shows the genes located in the selected locus. (**B**) 3D chromatin models reconstructed for the selected region for the reference cell line (left) and variant II (right) based on CTCF interactions (up) and CTCF + RNAPII interactions (down). (**C**) A responsive table of the distances between the NFKBIA gene and enhancers in the reference and variant. (**D**) Box plots of the distance distribution between the NFKBIA gene and enhancer located in the chr14:35046634–35048014 region for the reference and variant 2.

*Genome browser*. We have integrated the Interactive Genome Viewer (IGV) (17) as a genome browser, providing an alternative to presenting arc diagrams in static PNG format as in the previous version (Figure 2A). IGV is a highly responsive tool that allows users to visualise and manipulate genomic tracks, such as chromatin contact arcs, and gene, enhancer, and structural variation annotations for reference and variant samples. All data are displayed on the GRCh38 genome assembly. One notable feature of IGV is its ability to save results in vector file format (SVG), which makes it easy to present results outside the web server.

*3D viewer*. We have integrated NGL (18), a modern and interactive molecular visualisation tool, to present chromatin 3D structures dynamically and intuitively. NGL enables users to explore and interact with 3D models generated by 3D-GNOME, allowing them to adjust the view, zoom in and out, and rotate the structures to better understand the spatial relationships between genes and enhancers. Users can investigate the impact of structural variation on the 3D organisation of the genome by displaying both reference and variant 3D models on two separate 3D viewers, with coloured by genes and enhancers mapped on them (Figure 2B).

*Promoter–enhancer distance comparison*. We present the results of comparisons of promoter–enhancer distances in a responsive table generated using the Bootstrap package (Figure 2C). The table displays the genes, gene types (including pseudogenes), enhancers with an enhancer score, average gene-enhancer distance in an ensemble in the reference and variant structures, as well as differences between these two ensembles and p-values of the significance of those differences. Users can search, sort, and filter the results by columns. Furthermore, we have added an option to generate a distribution boxplot for a selected region (Figure 2D). The user may select rows with gene-enhancer pairs using checkboxes and use the ‘Generate distance boxplots’ button to submit the task. After that, using Ajax, the task is asynchronously transferred to Flask, the boxplots are calculated, and they are drawn using the Seaborn package (28).

Finally, after the automated page refreshing, the boxplots are viewed on the result web page. The boxplots with distances are displayed on the screen below the table and can also be downloaded from the download section.

*Download section*. The download section now includes the entire generated ensemble of models in mmCIF and XYZ format for manual analysis using common tools for visualising 3D structures, such as UCSC Chimera. Additionally, a *tsv* file with the results of the distance analysis is provided, including gene IDs, gene and enhancer coordinates, average distances in the ensemble, and the results of the Mann–Whitney *U* test of distribution changes (*P*-value and statistical value). Each gene-enhancer distance boxplot generated by clicking the ‘Generate boxplots’ button is also included in the output file folder.

## CONCLUSIONS AND FUTURE PLANS

This latest update to 3D-GNOME web server provides an advanced tool for analysing modelled distance changes between enhancers and gene promoters. This is a valuable resource for exploring the impact of 3D chromatin structure on gene transcription and regulation. The new version offers significantly improved speed and efficiency due to GPU acceleration and Eden cluster architecture, enabling faster and more efficient chromatin modelling and analysis. We have also added new tools, including the NGL Viewer and IGV genome browser, which enhance the user experience by providing an intuitive and visually appealing way to analyse data.

In the near future, we plan to extend our datasets of chromatin interactions by including additional cell lines, such as H1ESC, HFFC6 and WTC11, as well as new structure variants from the Simons Diversity Projects for modern humans and archaic populations, such as Neanderthals and Denisovans. Including these archaic populations will provide a unique opportunity to investigate the evolution of chromatin structure and its impact on gene regulation across different populations, shedding new light on the history and diversity of our species. We also plan to add new input formats and datasets, such as Hi-C data, which are already standard in the scientific community. To facilitate this, we plan to implement in the web server chromatin loop calling software, which is necessary for converting native Hi-C data for 3D-GNOME modelling. In the near future, we will add new annotation tracks to the IGV genome browser, such as cell line-specific H3K27Ac marks, and colour these genomic features on 3D models to improve accessibility and facilitate better analysis of complex interactions between them.

## DATA AVAILABILITY

3D-GNOME is freely available at https://3dgnome.mini.pw.edu.pl/.

## References

[1] Isbel L. , Grand R.S., Schübeler D. Generating specificity in genome regulation through transcription factor sensitivity to chromatin. Nat. Rev. Genet. 2022; 23:728–740. 35831531 10.1038/s41576-022-00512-6

[2] Hafner A. , Boettiger A. The spatial organization of transcriptional control. Nat. Rev. Genet. 2022; 24:1–16. 10.1038/s41576-022-00526-0 36104547

[3] Chiliński M. , Sengupta K., Plewczynski D. From dna human sequence to the chromatin higher order organisation and its biological meaning: using biomolecular interaction networks to understand the influence of structural variation on spatial genome organisation and its functional effect. Semin. Cell Dev. Biol. 2022; 121:171–185. 34429265 10.1016/j.semcdb.2021.08.007

[4] Farnham P.J. Insights from genomic profiling of transcription factors. Nat. Rev. Genet. 2009; 10:605–616. 19668247 10.1038/nrg2636 PMC2846386

[5] Heintzman N.D. , Ren B. Finding distal regulatory elements in the human genome. Curr. Opin. Genet. Dev. 2009; 19:541–549. 19854636 10.1016/j.gde.2009.09.006 PMC3321269

[6] Levine M. Transcriptional enhancers in animal development and evolution. Curr. Biol. 2010; 20:R754–R763. 20833320 10.1016/j.cub.2010.06.070 PMC4280268

[7] Schoenfelder S. , Fraser P. Long-range enhancer–promoter contacts in gene expression control. Nat. Rev. Genet. 2019; 20:437–455. 31086298 10.1038/s41576-019-0128-0

[8] Scott A.J. , Hall I.M., Chiang C Structural variants are a major source of gene expression differences in humans and often affect multiple nearby genes. Genome Res. 2021; 526:2249–2257. 10.1101/gr.275488.121 PMC8647827 34544830

[9] Gusev A. , Lee S.H., Trynka G., Finucane H., Vilhjálmsson B.J., Xu H., Zang C., Ripke S., Bulik-Sullivan B., Stahl E. et al. Partitioning heritability of regulatory and cell-type-specific variants across 11 common diseases. Am. J. Hum. Genet. 2014; 95:535–552. 25439723 10.1016/j.ajhg.2014.10.004 PMC4225595

[10] Maurano M.T. , Humbert R., Rynes E., Thurman R.E., Haugen E., Wang H., Reynolds A.P., Sandstrom R., Qu H., Brody J. et al. Systematic localization of common disease-associated variation in regulatory dna. Science. 2012; 337:1190–1195. 22955828 10.1126/science.1222794 PMC3771521

[11] Liu Y. , Qu H.-Q., Mentch F.D., Qu J., Chang X., Nguyen K., Tian L., Glessner J., Sleiman P.M., Hakonarson H. Application of deep learning algorithm on whole genome sequencing data uncovers structural variants associated with multiple mental disorders in african american patients. Mol. Psychiatry. 2022a; 27:1469–1478. 34997195 10.1038/s41380-021-01418-1 PMC9095459

[12] Liu Z. , Roberts R., Mercer T.R., Xu J., Sedlazeck F.J., Tong W. Towards accurate and reliable resolution of structural variants for clinical diagnosis. Genome Biol. 2022b; 23:68. 35241127 10.1186/s13059-022-02636-8 PMC8892125

[13] Wlasnowolski M. , Sadowski M., Czarnota T., Jodkowska K., Szalaj P., Tang Z., Ruan Y., Plewczynski D. 3D-GNOME 2.0: a three-dimensional genome modeling engine for predicting structural variation-driven alterations of chromatin spatial structure in the human genome. Nucleic Acids Res. 2020; 48:W170–W176. 32442297 10.1093/nar/gkaa388 PMC7319547

[14] Szalaj P. , Michalski P.J., Wróblewski P., Tang Z., Kadlof M., Mazzocco G., Ruan Y., Plewczynski D. 3D-GNOME: an integrated web service for structural modeling of the 3D genome. Nucleic Acids Res. 2016; 44:W288–W293. 27185892 10.1093/nar/gkw437 PMC4987952

[15] 1000 Genomes Project Consortium A global reference for human genetic variation. Nature. 2015; 526:68–74. 26432245 10.1038/nature15393 PMC4750478

[16] Szałaj P. , Tang Z., Michalski P., Pietal M.J., Luo O.J., Sadowski M., Li X., Radew K., Ruan Y., Plewczynski D. An integrated 3-dimensional genome modeling engine for data-driven simulation of spatial genome organization. Genome Res. 2016; 26:1697–1709. 27789526 10.1101/gr.205062.116 PMC5131821

[17] Thorvaldsdóttir H. , Robinson J.T., Mesirov J.P. Integrative Genomics Viewer (IGV): high-performance genomics data visualization and exploration. Brief. Bioinf. 2012; 14:178–192. 10.1093/bib/bbs017 PMC3603213 22517427

[18] Rose A.S. , Hildebrand P.W. NGL Viewer: a web application for molecular visualization. Nucleic Acids Res. 2015; 43:W576–W579. 25925569 10.1093/nar/gkv402 PMC4489237

[19] Kadlof M. , Rozycka J., Plewczynski D. Spring Model–chromatin modeling tool based on OpenMM. Methods. 2020; 181:62–69. 31790732 10.1016/j.ymeth.2019.11.014

[20] Wang Y. , Song F., Zhang B., Zhang L., Xu J., Kuang D., Li D., Choudhary M.N.K., Li Y., Hu M. et al. The 3D genome Browser: a web-based browser for visualizing 3D genome organization and long-range chromatin interactions. Genome Biol. 2018; 19:1–12. 30286773 10.1186/s13059-018-1519-9 PMC6172833

[21] Li X. , Luo O.J., Wang P., Zheng M., Wang D., Piecuch E., Zhu J.J., Tian S.Z., Tang Z., Li G. et al. Long-read ChIA-PET for base-pair-resolution mapping of haplotype-specific chromatin interactions. Nat. Protoc. 2017; 12:899–915. 28358394 10.1038/nprot.2017.012 PMC5537732

[22] Tang Z. , Luo O.J., Li X., Zheng M., Zhu J.J., Szalaj P., Trzaskoma P., Magalska A., Wlodarczyk J., Ruszczycki B. et al. CTCF-mediated human 3D genome architecture reveals chromatin topology for transcription. Cell. 2015; 163:1611–1627. 26686651 10.1016/j.cell.2015.11.024 PMC4734140

[23] Wang P. , Feng Y., Zhu K., Chai H., Chang Y., Yang X., Liu X., Shen C., Gega E., Lee B. et al. In situ chromatin interaction analysis using paired-end tag sequencing. Curr. Protoc. 2021; 1:e174. 34351700 10.1002/cpz1.174 PMC8351913

[24] Yoo A.B. , Jette M.A., Grondona M. Slurm: simple linux utility for resource management. Job Scheduling Strategies for Parallel Processing: 9th international Workshop, JSSPP 2003, Seattle, WA, USA, June 24, 2003. Revised Paper 9. 2003; Springer 44–60.

[25] Tange O. Gnu parallel-the command-line power tool. USENIX Mag. 2011; 36:42–47.

[26] Gao T. , Qian J. EnhancerAtlas 2.0: an updated resource with enhancer annotation in 586 tissue/cell types across nine species. Nucleic Acids Res. 2019; 48:D58–D64. 10.1093/nar/gkz980 PMC7145677 31740966

[27] Sadowski M. , Kraft A., Szalaj P., Wlasnowolski M., Tang Z., Ruan Y., Plewczynski D Spatial chromatin architecture alteration by structural variations in human genomes at the population scale. Genome Biol. 2019; 20:148. 31362752 10.1186/s13059-019-1728-x PMC6664780

[28] Waskom M.L. Seaborn: statistical data visualization. J. Open Source Softw. 2021; 6:3021.

